# Cardiotonic Steroids as Modulators of Neuroinflammation

**DOI:** 10.3389/fendo.2016.00010

**Published:** 2016-02-16

**Authors:** Ana Maria Orellana, Paula Fernanda Kinoshita, Jacqueline Alves Leite, Elisa Mitiko Kawamoto, Cristoforo Scavone

**Affiliations:** ^1^Department of Pharmacology, Institute of Biomedical Science, University of São Paulo, São Paulo, Brazil

**Keywords:** cardiotonic steroids, NKA, inflammation, immunomodulation, NF-κB, ouabain

## Abstract

Cardiotonic steroids (CTS) are a class of specific ligands of the Na^+^, K^+^- ATPase (NKA). NKA is a P-type ATPase that is ubiquitously expressed and although well known to be responsible for the maintenance of the cell electrochemical gradient through active transport, NKA can also act as a signal transducer in the presence of CTS. Inflammation, in addition to importantly driving organism defense and survival mechanisms, can also modulate NKA activity and memory formation, as well as being relevant to many chronic illnesses, neurodegenerative diseases, and mood disorders. The aim of the current review is to highlight the recent advances as to the role of CTS and NKA in inflammatory process, with a particular focus in the central nervous system.

## Introduction

Inflammation is an important contributor to the protection of the organism against injuries and in the maintenance of homeostasis (1), as well as being relevant in the central nervous system (CNS) to a number of key processes, including memory formation (2). However, in chronic and uncontrolled pathological conditions, such as diabetes (3), Alzheimer’s disease (4), and Parkinson’s disease (5), inflammatory processes can be detrimental. Inflammatory processes can modulate the activity of many different proteins, including Na^+^,K^+^-ATPase (NKA) (6), which is a conserved membrane protein that maintains the electrochemical gradient of the cell membrane using ATP as its energy source. Cardiotonic steroids (CTS) are natural ligands of NKA that can, in a dose-dependent manner, inhibit NKA pump activity (7) or trigger the activation of signaling pathways, such as Ras–Raf–MAPK and inositol trisphosphate receptor (InsP3R) activity, which can evoke calcium oscillations, in turn leading to the activation of the inflammation-associated transcription factor nuclear factor-kappa B (NF-κB) (8).

Numerous studies have shown CTS to modulate the function of many immune cells, both *in vitro* and *in vivo*, with effects *via* the inhibition of NF-κB and MAPK, thereby controlling some key cellular processes, such as proliferation and differentiation, as well as cytokine production (9–14).

This article reviews the literature addressing CTS effects in immune modulation, with a particular emphasis on effects in the CNS. This is of some importance as a growing body of data indicates a significant role for central inflammatory processes in the etiology and course of many neurodegenerative diseases and mood disorders.

## Na^+^,K^+^-ATPase: A Brief Description

For many years, NKA has aroused the interest of many groups in studying not only its pumping function, structure, and biochemistry but also, more recently, its role in signal transduction (15–17). NKA is well known to be responsible for the maintenance of ionic homeostasis where the hydrolysis of one molecule of ATP allows the establishment of an electrochemical gradient through the cellular efflux of three Na^+^ ions and an influx of two K^+^ ions. NKA is also important to the provision of cellular energy, through the cotransport of other substances, over the plasma membrane, given that many co- or counter-transporters use the Na^+^ and K^+^ ionic gradients (18). A further crucial role for the NKA is the re-establishment of the ionic gradient following neuronal action potential firing. This is due to the natural Na^+^ and K^+^ diffusion potential across the cell membrane, which establishes a steady state that is vital for all excitable tissues, and which needs to be repolarized after neuronal depolarization (15, 18).

NKA consists of two protomers, each is composed of α- and β-subunits associated with a third protein, the γ-subunit or FXYD2 (18). Four different isoforms of the NKA α-subunit have been identified (α1–4), three β isoforms (β1–3), and seven different FXYD accessory proteins (19). Interestingly, the distribution of the different isoforms varies according to cell type, tissue, stage of development, and species (19–21). The isoforms α1, α2, α3, and α4 have different expression profiles, with the α1 subunit being ubiquitously expressed and uniformly distributed, α2 being predominant in some specific cell types, including astrocytes and cardiac myocytes, α3 being more highly expressed in neurons and the ovaries, while α4 can be found only in spermatozoa (15, 19, 22, 23). The NKA β-subunit seems to be a specific chaperone that is essential for both the traffic and correct plasma insertion site of newly synthesized α-subunits (24). Furthermore, the β-subunit can even influence pump activity, given its modulation of K^+^ affinity for the pump binding sites (25, 26). Interestingly, β1 and β2 isoforms can both be found in the brain, with astrocytes expressing α1 and α2 in combination with β2, while neurons express α1 and α3 in combination with β1 or β2 (15, 27).

## Importance of NKA Subunits in Inflammation: A General Approach

Over the last decade, many studies have suggested an interesting relationship between inflammatory process and the different NKA subunits, the main points of which will be summarized here.

It is well established that mitogenic activation of human peripheral blood lymphocytes by phytohemagglutinin (PHA) is accompanied by an enhancement of NKA activity, followed by an increase in K^+^ and Na^+^ transport (28, 29). This is dependent on new protein transcription (30, 31), and follows α1 and β1 mRNA up-regulation (32). In 2002, Chiampanichayakul et al. showed for the first time that an antibody anti-NKA β3 subunit was able to down-regulate the proliferation of both T- and B-lymphocytes, as well as the production of interleukin (IL)-2, IL-4, IL-10, and interferon-gamma (IFN-γ), *in vitro* (33).

In a model of systemic inflammation, male Sprague-Dawley rats that received an injection of Complete Freund’s adjuvant, which includes inactivated *Mycobacterium tuberculosis*, presented both α1- and α3-NKA isoform up-regulations, especially in primary afferent neurons, after 23 and 24 h (34). Furthermore, a recent study has brought some new insight as to how NKA may be involved in inflammatory processes in the somatosensory system (35). This study suggested that the FXYD2 subunit interaction with α1-subunit in murine nociceptive neurons negatively regulated pump activity, leading to membrane potential depolarization of these neurons, which can facilitate excitatory afferent neurotransmission, thereby facilitating allodynia. When the group used FXYD2-deficient mice, allodynia was more quickly abolished (35).

In general, it seems that the differences in the NKA isoform functions and distribution contribute somehow to the complexity of the inflammatory responses.

In 4-month-old rat hippocampus, the systemic injection of lipopolysaccharide (LPS) decreased both total NKA activity, and α2 and α3 isoforms, although in adult rats there was no significant difference (36). In accordance with these findings, *in vitro* experiments showed that inflammation induced by LPS-treated primary astrocytes co-cultured with newborn cortex endothelial cells also involves NKA downregulation (37). An LPS-dependent decrease in total NKA activity in young rats can be rescued by intermittent fasting (IF), as well as in older rats to a lesser degree (36), suggesting an age-associated susceptibility of NKA activity modulation by inflammatory process.

**Figure 1:**
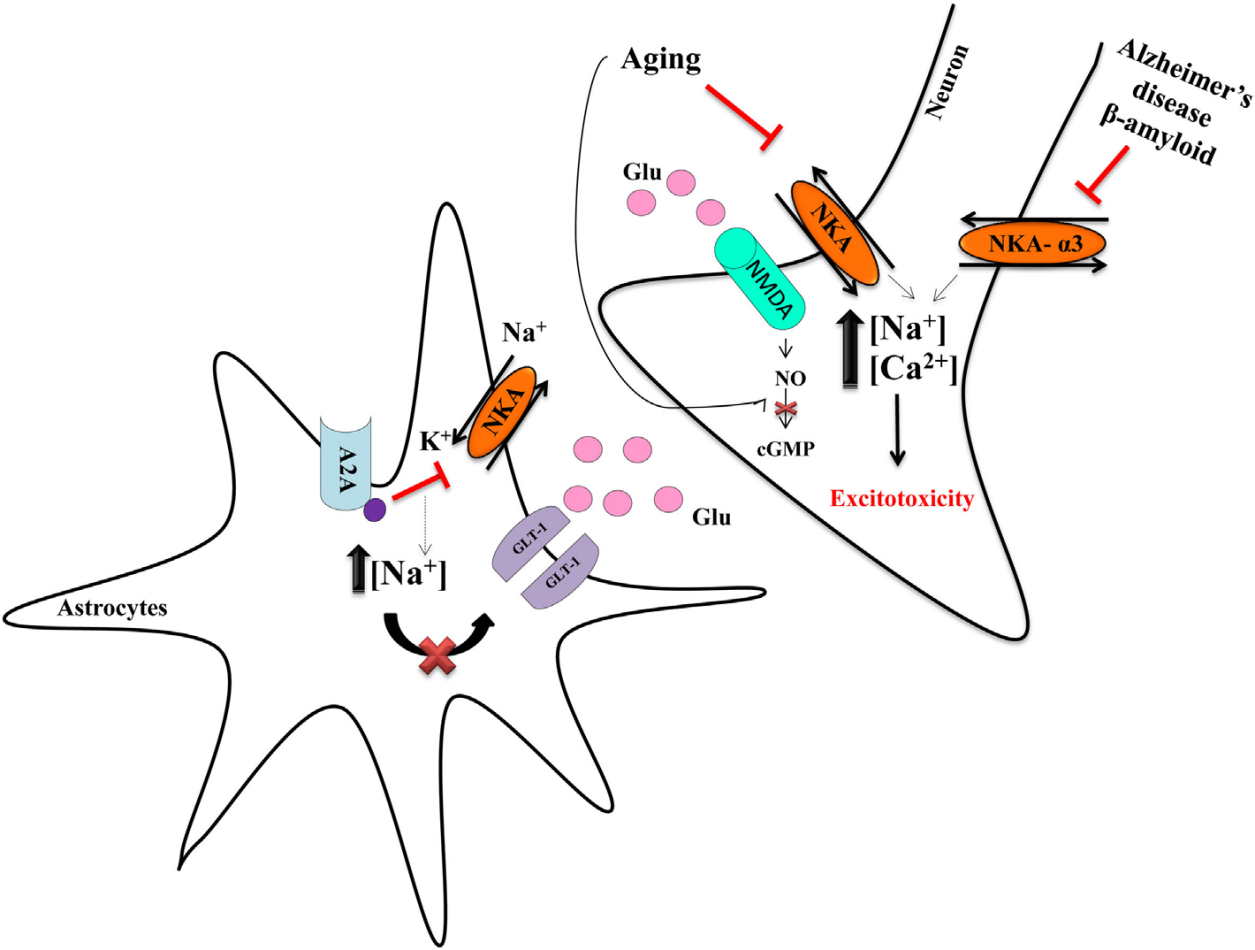
**Neurodegeneration and NKA**. Astrocytes are important to regulate the release of glutamate and its provision to neurons. NKA has an important role in the control of the glutamate transporter-1 (GLT-1) in astrocytes, because the concentration of Na^+^ modulates the uptake of glutamate (55). The adenosine A2A receptor decreases NKA activity, leading to increased intracellular Na^+^ concentration, which impairs glutamate uptake, resulting in more glutamate availability in the synaptic cleft (55). Such excess of glutamate overactivates the NMDA receptor, leading to increase intracellular Na^+^ and Ca^2+^, in turn driving excitotoxicity (55). The decrease of NKA is mostly deleterious and amylospheroids, which are composed of β-amyloid oligomers and α-synuclein, interact with α3-NKA, thereby impairing its activity. Such data indicate NKA to have an important role in neurodegeneration and neurodegenerative diseases (52, 53). Our group showed that aging is also an important factor that decreases NKA activity (36, 38).

However, in the rat cerebellum, there is an age-related decline of NKA activity, arising from a decrease in the cyclic GMP–PKG pathway in cerebellum. There is also an effect of glutamatergic activity in the modulation of alpha 2,3-NKA due to impairments in PKG signaling. A failure in the correction of ionic disturbances mechanism present in aging processes could also increase the probability of occurrence of degenerative disorders (38). As such, NKA activity can be modulated by different stimuli, with consequences for the neuro-inflammatory processes that are present in all neurodegenerative diseases (39) (Figure 1).

## NKA and Neuroinflammation

In the CNS, inflammatory processes utilize a wide array of immunological cells, including the resident immunological cells, microglia, and astrocytes (40). However, in a pathological situation, monocyte-derived macrophages, present in the perivascular space, are able to cross the blood–brain barrier (BBB), with lymphocytes also being able to transverse the epithelial blood–cerebrospinal fluid barrier (BCSFB) surrounding the choroid plexus (41–43).

When exposed to cytokines, innate immune modulators, hypoxia, or other damaging stimuli, astrocytes are able to release cytokines and other immune signaling molecules (40, 44), followed by changes in Ca^2+^ signaling system and Na^+^ transports during inflammation (37). Little can be stated generally about the microglial response, due to inflammation heterogeneity and complexity in different pathological contexts (45).

It is important to note that NKA plays a significant role in central inflammation, which is apparent in diabetic rats, where inflammation-associated increases in tumor necrosis factor (TNF)-α and IL-1β, as well as decreased NKA activity, result in memory impairment (46). Similarly, decreased NKA activity is evident in a model of traumatic brain injury (TBI) (47), which is prevented by exercise pre-conditioning, indicating that modulating NKA can determine the brain’s subsequent response to damage (6).

Differences between brain region responses during systemic inflammation have been found in an animal model of sepsis, with NKA inhibition being faster in the cortex versus the hippocampus (48). This effect is not due to differences in NKA mRNA levels or in NKA isoforms, but could be due to a post-translational modification or to an altered oxidative stress response. Only in the cortex does an increase in NKA activity follow antioxidant administration, suggesting that variations in oxidative status may play a more important role in the regulation of cortical NKA and thereby have a differential effect on the cortex inflammatory response versus that in the hippocampus (48). The hippocampus seems more vulnerable to inflammation and, at least partly *via* differential NKA antioxidant effects, is more vulnerable in neurodegenerative diseases, including Alzheimer’s disease (49).

In postmortem brains of Alzheimer’s disease patients, NKA activity is diminished compared to that of age-matched normal brains (50). Such results were reproduced in an *in vitro* model of hippocampal neurons treated with β-amyloid (51), as well as in an *in vitro* model of frontal cortex neurons, where the addition of antioxidants, such as genistein, afforded a protective effect by increasing NKA activity (50). The mechanism underlying such decreased NKA activity was recently proposed. The amylospheroids, amyloid β-protein (Aβ) oligomers typically found in postmortem brains of Alzheimer’s disease patients, can interact with α3-NKA impairing its activity, in turn leading to the activation of voltage-dependent calcium channels, mitochondrial abnormalities, and neurodegeneration (52) (Figure 1).

In addition, an increased sodium concentration in frontal and parietal cortex, as well as an increased potassium concentration in the cerebellar tissue of Alzheimer’s disease patients has also been shown. The same pattern appears in the cerebrospinal fluid (CSF) and was also replicated *in vitro* when astrocytes were treated with β-amyloid protein. In fact, Alzheimer’s disease is also related to impaired glutamate clearance, which contributes to an ionic imbalance (53).

During inflammation, an increase in *N*-methyl d-aspartate (NMDA) receptor phosphorylation is observed, with increased IL-1β release from astrocytes also evident (54). When a co-culture model of astrocytes pre-activated by an inflammatory stimulus was treated with Ifenprodil, Lundborg and colleagues observed that this drug could be a potent anti-inflammatory in astrocytes, following its restoration of Ca^2+^ levels and increased NKA expression (54).

In astrocytes, there is a crosstalk between α2-NKA isoform and GLT-I, an astrocytic glutamate transporter, that seems to be regulated by the Na^+^ pump (55) (Figure 1). Furthermore, adenosine A2A receptors (A2AR), present in astrocytes, have an important role in plasticity and neurodegeneration (55). The activation of these receptors decreases NKA activity, which, as a result, inhibits glutamate uptake. In a model of A2AR knockout animals, increased astrocytic glutamate uptake occurred, coupled to increased expression of α2-NKA in the cortex and striatum. This suggests a role for NKA in neurodegeneration (55), including Parkinson’s disease, given that in 2004, it was discovered that α3-NKA mutations are involved in rapid-onset dystonia parkinsonism that reveals that NKA could have an important role in Parkinson’s disease (56). Of note, extracellular α-synuclein can interact with α3-NKA to form clusters in the membrane of neurons that reduce the extrusion of Na^+^ and which may be a relevant neuropathological change in Parkinson’s disease (57). In addition, there is also evidence that α1-NKA overexpression drives axon initial segment abnormalities and that by reducing its expression, these and other phenotypes could be corrected in an Angelman syndrome murine model (58). The lack of NKA is involved in neurodegeneration given that α-NKA deletion in *Drosophila* photoreceptors lead to the loss of visual function, as well as an increase in neurodegeneration (59).

## Cardiotonic Steroids, the Specific Ligands of NKA

Cardiotonic steroids are a class of compounds that have been used to treat congestive heart failure since the eighteenth century (60). These compounds bind specifically to NKA, inhibiting its activity in a dose-dependent manner. Furthermore, they can also activate wider cellular signaling pathways, mediating changes in intracellular Ca^2+^ homeostasis, control of cellular growth, and gene expression (19, 61–64).

Cardiotonic steroids can be divided into cardenolides and bufadienolides, according to their origins (7) and molecular structure (19, 65, 66), being subsequently classified as endogenous hormones in the 1990s. Two cardenolides, digoxin (67) and ouabain (68), and several bufadienolides (69–71) have been identified in human tissues. They are proposed to be produced in the hypothalamus and adrenal gland (19, 64, 72), although there is some controversy regarding this, as some work has failed to show their presence in human plasma (73).

Numerous studies have shown CTS, as natural ligands of NKA, to have modulatory effects on immune cells functioning *in vitro* and *in vivo* (9–11, 13, 14).

## Anti-Inflammatory Properties of CTS

In 1997, Matsumori et al. demonstrated that ouabain reduced the production of the pro-inflammatory cytokines, IL-6 and TNF-α, in LPS-stimulated human peripheral blood mononuclear cells, as similarly observed *in vivo*, in LPS-treated mice (12). Following this work, other studies have demonstrated anti-inflammatory and analgesic effects of CTS in mice. In the classical paw edema model, pretreatment with ouabain reduced carrageenan-, zymosan-, 48/80-, and PGE2-induced edema and vascular permeability (14, 74), with bufalin leading to a reduction in carrageenan-induced paw edema, due to the inhibition of NF-κB activation and reduced pro-inflammatory protein expression, including inducible nitric oxide synthase (iNOS), cyclooxygenase-2, TNF-α, IL-1β, and IL-6 (13). Furthermore, bufalin has an antinociceptive effect, due, at least in part, to its reduction of TNF-α and IL-1β release (13). Moreover, bufalin inhibits inflammation and migration of TNF-α-induced fibroblast-like synoviocytes, as well as reducing IL-1β, IL-6, IL-8 expression and decreasing the secretion of matrix metalloproteinase-9 (MMP-9), driven by its suppression of NF-κB activation. These results suggest a potential modulatory effect of bufalin in diseases, such as rheumatoid arthritis (75).

Similar to bufalin, ouabain has the ability to reduce pro-inflammatory cytokine production, such as IL-1β and TNF-α, likewise inhibiting NF-κB and p38 MAPK signaling pathways (11, 14) as demonstrated in a study showing that ouabain decreases intraperitoneal polymorphonuclear cell concentrations induced by several stimuli, as well as inhibiting leukocyte migration *in vitro* (14, 74, 76). A similar anti-inflammatory effect was also seen with oleandrin and digitoxin (77, 78).

Recently, a subset of IL-17-producing cells has been linked to chronic inflammatory processes (79). These cells are described as a new population of effector T cells, known as Th17 cells (80), and are characterized by retinoic acid orphan receptor (ROR)-γt transcription factor and by IL-17A and IL-17F production (81), but not T-bet or GATA-3 transcription factors (82). Several studies have investigated digoxin effects in Th17 cells (83–85). Treatment with digoxin, in a mouse and human CD4^+^ T cell culture, inhibited naïve CD4 polarization to Th17 cells and reduced IL-17A protein expression. These effects were due to the inhibition of RORγt transcriptional activity. Furthermore, in experimental autoimmune encephalomyelitis, a multiple sclerosis model and Th17-mediated disease, treatment with digoxin, delays disease onset and reduces disease severity. IL-17 levels produced by infiltrating cells were also decreased (83).

Taken together, numerous studies have shown CTS to modulate the function of immune cells *in vitro* and *in vivo*. These anti-inflammatory effects are mainly mediated through NF-κB and MAPK inhibition. Besides CTS effects in the periphery as indicated above, some studies have also been carried out in the CNS, although the exact mechanisms or signaling pathways involved are still poorly understood. In the next section, studies are described that evaluate the functions of CTS in neuroinflammation.

## CTS and Immune Response in the CNS

Neuroinflammation is currently a hot topic, primarily due to its association with neurodegenerative diseases, such as Parkinson’s disease and Alzheimer’s disease, as well as its association with mood disorders and cancers. As such, neuroinflammation has become a highly significant therapeutic target (86).

Cardiotonic steroids can have contrasting effects on neuroinflammation, as determined by variations in concentration, the route of administration, cell type, specific brain region, and time course. Ouabain is one of the most studied CTS in the CNS.

Although ouabain-like compounds are supposed to be produced in the hypothalamus (47), there has been little investigation as to its central effects, including the regulation of central inflammatory responses and whether the CTS are able to cross the BBB by getting CNS entry *via* the fenestrated vasculature adjacent to the circumventricular organs (87).

Many groups have reported that low concentrations of this glycoside did not inhibit NKA, instead it could activate signaling pathways that usually result in a protective response, by increasing anti-apoptotic protein expression and cell proliferation (88–90). When the cells are treated with higher concentrations of ouabain (micrometer–millimeter), NKA is inhibited and, as a consequence, Na^+^ and K^+^ gradients are lost leading to an influx of Ca^2+^ into the cell, which can drive neuronal excitotoxicity (91).

In a rat cerebellum cell culture treated with ouabain at 10 μM, an increase in NF-κB nuclear translocation was observed, thereby increasing the mRNA levels of *Tnf-*α*, Il-1*β, and *Bdnf*. In addition, the same study showed that ouabain-induced NF-κB activation was dependent on the NMDA–Src–Ras signaling trigged by the MAPK pathways (91). Similar results were observed in another study from the same group, in a model of intra-hippocampal injections of 10 nM ouabain concentrations in adult rats. At nanomolar concentration, no inhibition of the NKA activity was observed, and there was NF-κB activation followed by an increase of *Tnf-*α and *iNos* mRNA levels (92). These two studies show that ouabain could increase pro-inflammatory cytokines without NKA inhibition and that NF-κB, Src, and NMDA are involved in this signaling pathway. As such, ouabain could also play an important role in the control of NF-κB activation, which can lead to adaptive responses and this could have an important impact on hippocampal memory processes, including memory formation and/or consolidation (93).

**Figure 2:**
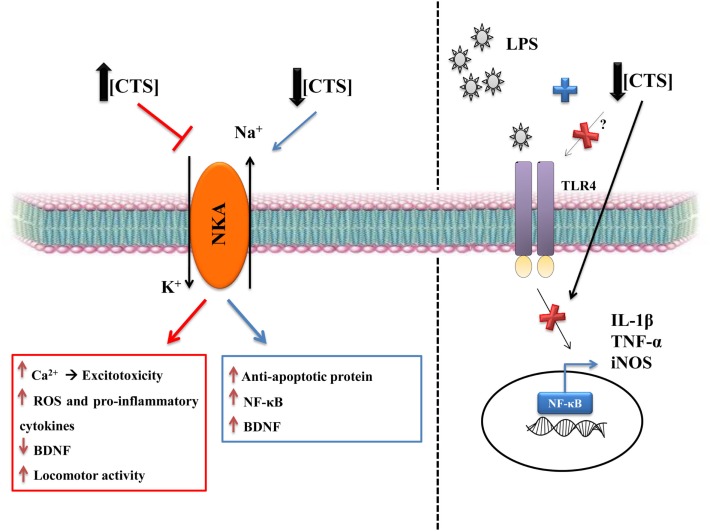
**The dual role of CTS**. CTS at high concentrations are deleterious, due to NKA inhibition that consequently increases intracellular Na^+^, which leads to raised intracellular Ca^2+^ that can be the first signal of neuronal excitotoxicity (91). There is also an increase in reactive oxygen species and in pro-inflammatory cytokines and a decrease in BDNF, which is a relevant growth factor in the brain (108, 109, 114). CTS also increase locomotor activity (114), particularly when the CTS are intracerebroventricular injected, as in the ouabain model of mania (110). CTS seem to be involved in mood disorders, such as depression and bipolar disorder. Low concentrations of CTS can have a protective role, including the CNS. CTS can activate anti-apoptotic protein, such as Bcl-xL, as well as increasing NF-κB and BDNF (88–90, 100). Our group showed that the pretreatment with ouabain can be protective against neuroinflammation caused by LPS (90). This protection is due to decreased IL-1β, TNF-α, and iNOS mRNA levels and in the rescue of BDNF levels, thereby reverting the changes induced by LPS and, consequently, decreasing astrocytic activity, as measured by dentate gyrus GFAP (90).

In addition, a third study from the group showed that ouabain can also play a protective role against LPS, a LPS that causes systemic immune responses and can also disrupt the BBB at higher concentrations. In these experiments, rats were pretreated with a very low concentration (1.8 μg/kg) of ouabain intraperitoneally before LPS. Results suggested that ouabain could decrease the nuclear translocation of p65, a NF-κB subunit, important in inflammatory responses, in the presence of LPS and also reversed LPS-induced decrease in cytoplasmic IκB levels. As a result, *Il-1*β and *iNos* gene expression were decreased in ouabain pretreatment, in comparison to the LPS-only group (90) (Figure 2).

As previously described, ouabain can also increase TNF-α release, but when animals were treated with ouabain and then challenged with LPS, there was a decrease in the levels of this cytokine. This anti-inflammatory property of ouabain was also seen in the dentate gyrus, a region of the hippocampus, with ouabain decreasing astrocyte activity in comparison to the LPS-only group (90). Such data indicate that ouabain can also be protective, during disturbances to CNS homeostasis, suggesting that ouabain alone in physiological concentrations may modulate cellular homeostasis, *via* pro-inflammatory cytokine regulation. It also suggests that the signaling pathway activated by ouabain could be an interesting target in neuroinflammation-associated disorders.

Interestingly, two separate studies noted that different cell types have specific ouabain responses. In rat cortex astrocyte culture, ouabain could prevent the downregulation of NKA and also could attenuate the raised levels of IL-1β release induced by LPS (94). However, in microglia, ouabain in low and high concentrations did not have any effect against LPS-induced cytokine release, although low concentration of ouabain evoked an increase in TNF-α release, which highlights that ouabain can have differential immune-modulatory effects in different cell types (95).

Furthermore, Kaur et al. have also observed that digoxin seems to have a neuroprotective effect in an ischemia/reperfusion model (96). Although, the authors did not test any inflammation parameter, the immune system is activated in this protocol with digoxin pretreatment decreasing the cerebral infarct size, resulting in better motor behavior and memory. These authors used Ca^2+^ channel blockers, which abolished this protection, suggesting efficacy *via* the Ca^2+^ signaling cascades (96). Another way that digoxin can be protective is by increasing levels of endogenous brain hydrogen sulfide (H_2_S) (97). H_2_S is severely decreased in the brain of Alzheimer’s disease patients (98), suggesting that this could be another mechanism by which CTS could alleviate symptomatology in patients with neurodegenerative diseases, although the concentration may have to be very low.

Although the discussion of CTS effects on glutamatergic excitotoxicity is beyond the scope of this review, some data are worth highlighting. Ouabain, at 1 nM, seems also to be protective against kainate-induced neurotoxicity by preventing Ca^2+^ overload in the rat cortical neurons, indicating that the modulation of NKA can play a key role against neuronal degeneration (88). The same effect seems to occur in an experimental model of ischemia in hippocampal slice cultures, where ouabain at 120 nM increases NKA activity. NKA inhibition is important to this ouabain-mediated protective effect, which suggest that low dose of ouabain and digoxin may be a plausible and novel treatment strategy in the management of stroke (39).

Another study showed the protective effect of oleandrin in an ischemia model *in vitro* and *in vivo*, suggesting that the protective effect of CTS in low doses is common to all types of CTS (99). It seems that oleandrin increased plasma brain-derived neurotrophic factor (BDNF) levels, this being another possible mechanism of protection against oxygen-glucose deprivation (100).

Neriifolin, another CTS, was used as a model of Parkinson’s disease, because it decreases dopaminergic neuron survival through NKA inhibition, which increases the concentration of intracellular Na^+^, reactive oxygen species (ROS), and p53 activation (101). Similarly, marinobufagenin (MGB) can also induce endothelial oxidative stress, which may be the first step in the emergence of a stroke, as well as being of relevance in dementias, including Alzheimer’s disease (102).

## The Inflammatory Background in Mood Disorders and CTS

Accumulating evidence has shown that there is a strong correlation between mood disorders and elevated levels of CSF cytokines, such as TNF-α and IL-1β, as well as increased PGE2 levels (103). Thus, it has been suggested that NKA activity and its modulation may also have a pertinent role in the etiology of mental disorders, especially bipolar disorder, which is associated with NKA activity abnormalities in erythrocytes (104) and high peripheral levels of pro-inflammatory cytokines (105).

In samples of bipolar disorders patients, ouabain binding to cortex synaptosomal preparations is lower when compared to depressed and schizophrenic groups and, at the same time, the levels of ouabain were higher in the parietal cortex from these patients in comparison with the other groups studied (106). However, this difference was not a consequence of changes in the expression of the α-NKA isoforms. In the same study, LPS was used as a rodent model for depression, leading to an increase in the level of ouabain-like compounds in the adrenal gland (106).

However, when animals were treated with anti-ouabain antibody or a low dose of ouabain, the depressive behaviors caused by LPS was reverted. Although intriguing, the results are complex, given that some similar effects were found from the antibody and ouabain treatments. First, endogenous ouabain-like compounds respond differently from exogenous ouabain; second, different CTS can have different physiological outcomes (107) or the use of the antibody could have a positive feedback on levels of endogenous ouabain-like compounds produced in the brain, leading to a similar effect to that of exogenous ouabain (106).

Ouabain, at a high concentration (1 mM), is also used as a model of mania, due to NKA inhibition, with this inhibition correlating with the increased cytokine levels, which are also evident in bipolar patients (108), as well with the decreased BDNF levels in hippocampus and amygdala in this rat model (109). Another important feature of ouabain in this model of mania is locomotor hyperactivity, reduced by acute or chronic treatment with memantine, lithium (110), or valproate (109). Memantine is a well-known NMDA receptor blocker (111), first indicated for the treatment of moderate-to-severe Alzheimer’s disease, but that has been used off-label to treat various psychiatric disorders, including as a mood stabilizer (112). Although the mechanisms by which lithium antagonizes ouabain are not clear, it was observed in rat hippocampal slices that ouabain (3.3 mM) treatment induces cycling electrically evoked epileptiform responses, which is delayed by pretreatment with lithium (113).

Another aspect of hyperlocomotion, including the rat ouabain model of mania, is increased levels of ROS and protein carbonyl levels, as well as the inhibition of catalase and glutathione peroxidase (114). The involvement of oxidative stress in mania was also shown following the intracerebroventricular (ICV) injection of ouabain (1–10 mM), which increased thiobarbituric acid-reactive substances (TBARS) levels and superoxide in the rat amygdala, cortex, and striatum (115).

In addition, at high concentration, ouabain ICV injection (10 mM) modulates NKA by increasing the expression of α2-NKA in glial cells of the basal ganglia and α3-NKA in neuron of the frontal cortex, which may be related to hyperactivity behavior. When compared to human postmortem studies, a decrease of α2-NKA expression in temporal cortex of bipolar disorder patients was observed, suggesting that the specific NKA isoform expression levels may be important to the pathophysiology of mania (116).

Another interesting finding is that endogenous production of CTS in bipolar disorder patients also seems to be impaired. Bipolar disorder patients have less digoxin-like immunoreactive factor in comparison to controls, with this low level not being season dependent, as in the case of controls, where winter levels are lower than during the rest of the year (117). The ouabain-like immunoreactive factor (OLF) is also lower in bipolar disorder patients. During physical exercise, OLF is increased in non-psychiatric patients, but in bipolar disorder patients OLF levels tend to be lower, even during exercise. As such, the regulation of OLF seems impaired in bipolar disorder patients (118).

A meta-analysis of 30 studies with a total of 2,599 participants showed evidence for a significant increase in pro-inflammatory and regulatory cytokines in bipolar disorder patients (119). However, despite ICV injection of ouabain 10 mM in rats mimicking some aspects of bipolar disorder, ouabain injection does not increase the inflammatory molecules that are present in bipolar disorder patients, with ICV injection of high-dose OUA decreasing striatal IL-6 levels (108).

## Conclusion and Future Perspective

It is clear that NKA is modulated by and modulates inflammatory process through its different inflammatory molecules, with this occurring in many different ways according to cell type, tissue, age, and the dose of its specific CTS ligands, suggesting a complex role for CTS in inflammatory processes. In the CNS, inflammation has been suggested as an important mediator in major neurodegenerative diseases. As such, the role of CTS and NKA in inflammatory process provides a significant challenge for future research, including as to the role of endogenous CTS and in the modulation of NKA levels and activity, which is fundamental for the optimal functioning of the synaptic activity of the nervous system. By virtue of its involvement in such fundamental processes, the investigation of CTS is likely to drive the development of novel therapeutic interventions.

## Author Contributions

Conceived and designed the manuscript: EK and CS. Wrote the manuscript: AO, PK, and JL have contributed equally to this work. Final revision: EK, CS, AO, PK, and JL.

## Conflict of Interest Statement

The authors declare that the research was conducted in the absence of any commercial or financial relationships that could be construed as a potential conflict of interest.
